# Dichlorido(dimethyl­formamide-κ*O*)[1,4,7-tris­(2-cyano­ethyl)-1,4,7-triaza­cyclo­nonane-κ^3^ *N* ^1^,*N* ^4^,*N* ^7^]nickel(II)

**DOI:** 10.1107/S1600536808024422

**Published:** 2008-08-06

**Authors:** Zhong Zhang, Li-Zhen Wu, Zhi-Rong Geng, Zhi-Lin Wang

**Affiliations:** a Coordination Chemistry Institute, State Key Laboratory of Coordination Chemistry, Nanjing University, Nanjing 210093, People’s Republic of China

## Abstract

The title complex, [NiCl_2_(C_15_H_24_N_6_)(C_3_H_7_NO)], is isomorphous with the Co^II^ analogue. Three N-atom donors from the facially coordinating triaza macrocyclic ligand, one O-atom donor from dimethyl­formamide and two Cl^−^ anions surround the Ni^II^ ion in a distorted octa­hedral coordination geometry. Inter­molecular C—H⋯Cl and C—H⋯N hydrogen-bonding inter­actions link the complex mol­ecules into a three-dimensional supra­molecular architecture.

## Related literature

For related literature, see: Graham *et al.* (2005 ▶); Li *et al.* (2005 ▶); Schlager *et al.* (1995 ▶); Tei *et al.* (1998 ▶ and 2003 ▶). For the isostructural Co complex, see: Zhang *et al.* (2008 ▶).
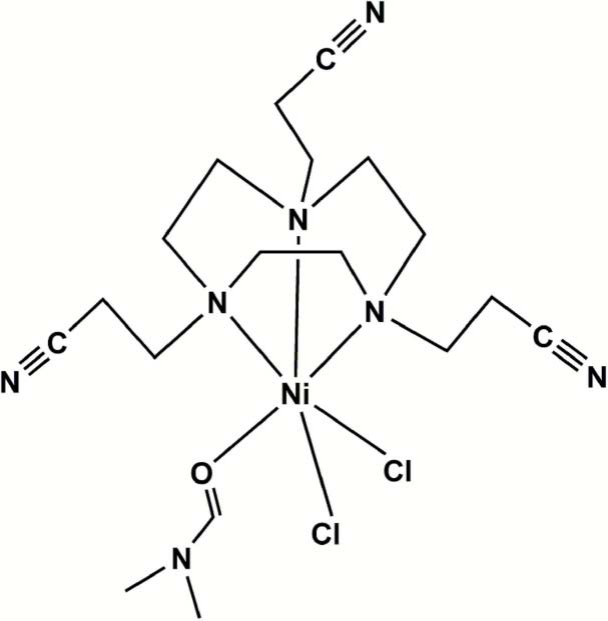


## Experimental

### 

#### Crystal data

[NiCl_2_(C_15_H_24_N_6_)(C_3_H_7_NO)]
*M* *_r_* = 491.11
Monoclinic, 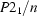

*a* = 9.7657 (10) Å
*b* = 19.698 (2) Å
*c* = 12.3504 (13) Å
β = 97.676 (2)°
*V* = 2354.5 (4) Å^3^
*Z* = 4
Mo *K*α radiation
μ = 1.07 mm^−1^
*T* = 298 (2) K
0.32 × 0.24 × 0.22 mm

#### Data collection

Bruker SMART APEX CCD area-detector diffractometer
Absorption correction: multi-scan (*SADABS*; Bruker, 2000 ▶) *T*_min_ = 0.725, *T*_max_ = 0.798
12684 measured reflections
4616 independent reflections
3371 reflections with *I* > 2σ(*I*)
*R*_int_ = 0.050

#### Refinement

*R*[*F* ^2^ > 2σ(*F* ^2^)] = 0.053
*wR*(*F* ^2^) = 0.115
*S* = 1.00
4616 reflections
265 parameters
H-atom parameters constrained
Δρ_max_ = 0.32 e Å^−3^
Δρ_min_ = −0.48 e Å^−3^

### 

Data collection: *SMART* (Bruker, 2000 ▶); cell refinement: *SAINT* (Bruker, 2000 ▶); data reduction: *SAINT*; program(s) used to solve structure: *SHELXTL* (Sheldrick, 2008 ▶); program(s) used to refine structure: *SHELXTL*; molecular graphics: *SHELXTL* and *ORTEP-3* (Farrugia, 1997 ▶); software used to prepare material for publication: *SHELXTL*.

## Supplementary Material

Crystal structure: contains datablocks I, global. DOI: 10.1107/S1600536808024422/bh2184sup1.cif

Structure factors: contains datablocks I. DOI: 10.1107/S1600536808024422/bh2184Isup2.hkl

Additional supplementary materials:  crystallographic information; 3D view; checkCIF report

## Figures and Tables

**Table:** 

Cl1—Ni1	2.4315 (10)
Cl2—Ni1	2.4158 (10)
N1—Ni1	2.180 (3)
N2—Ni1	2.134 (3)
N3—Ni1	2.144 (3)
Ni1—O1	2.093 (2)

**Table:** 

O1—Ni1—N2	88.31 (10)
O1—Ni1—N3	170.81 (11)
N2—Ni1—N3	83.12 (11)
O1—Ni1—N1	92.12 (10)
N2—Ni1—N1	83.15 (11)
N3—Ni1—N1	83.59 (11)
O1—Ni1—Cl2	89.34 (7)
N2—Ni1—Cl2	175.06 (8)
N3—Ni1—Cl2	98.94 (8)
N1—Ni1—Cl2	92.60 (8)
O1—Ni1—Cl1	90.90 (7)
N2—Ni1—Cl1	93.35 (8)
N3—Ni1—Cl1	92.91 (8)
N1—Ni1—Cl1	175.31 (8)
Cl2—Ni1—Cl1	91.02 (3)

**Table 2:** Hydrogen-bond geometry (Å, °)

*D*—H⋯*A*	*D*—H	H⋯*A*	*D*⋯*A*	*D*—H⋯*A*
C1—H1*A*⋯Cl2^i^	0.97	2.76	3.667 (4)	156
C3—H3*A*⋯Cl2^i^	0.97	2.80	3.756 (4)	168
C11—H11*A*⋯Cl1^i^	0.97	2.66	3.565 (4)	155
C11—H11*B*⋯Cl2^ii^	0.97	2.65	3.497 (4)	146
C18—H18*B*⋯N5^iii^	0.96	2.55	3.445 (7)	155

Symmetry codes: (i) 
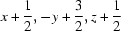
; (ii) 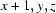; (iii) 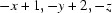.

**Fig. 1.**
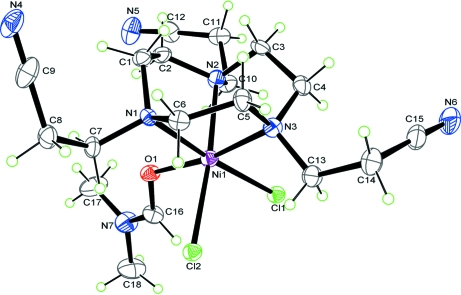
The molecular structure of the title compound, showing displacement ellipsoids at the 30% probability level (arbitrary radii spheres for H atoms).

**Fig. 2.**
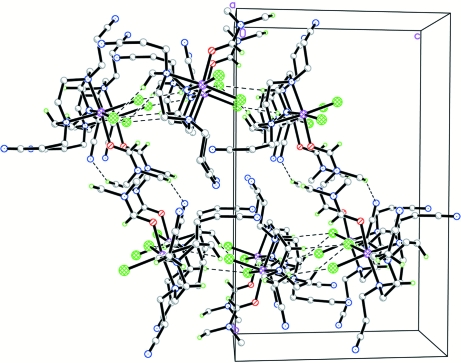
Packing diagram of the title compound, showing the three-dimensional network formed through intermolecular C—H···Cl and C—H···N hydrogen bonds (dashed lines). For clarity, H atoms not involved in hydrogen bonding have been omitted.

## supplementary crystallographic information

### Comment

1,4,7-Triazacyclononane ([9]aneN_3_) derivatives with nitrile pendant arms have attracted much interest since these triazamacrocyclic ligands can promote the assembly of multi-dimensional polymeric compounds with Ag^I^ (Tei *et al.*, 1998). However, these triazamacrocyclic ligands exhibit different coordination behaviors when coordinating to Cu^II^ and only mononuclear Cu^II^ complexes of these ligands can be obtained, where the nitrile pendant arms are not involved in the metal coordination (Tei *et al.*, 2003). Herein, we report the synthesis and crystal structure of a monomeric Ni^II^ complex containing 1,4,7-tris(2-cyanoethyl)-1,4,7-triazacyclononane, (I), which is isostructural to its cobalt-containing analogue (Zhang *et al.*, 2008).

As depicted in Fig. 1, the Ni^II^ ion in this complex is ligated by a [N_3_OCl_2_] donor set consisting of three N atoms from the [9]aneN_3_ backbone, an O atom from a dimethylformamide molecule and two Cl^-^ anions. The twist angle is *ca*. 57.6° (Schlager *et al.* 1995), indicating that the coordination geometry around Ni^II^ is slightly distorted from regular octahedral. All bond lengths around Ni^II^ ion (Table 1) are comparable to those observed in related Ni^II^ complexes (Graham *et al.* 2005; Li *et al.* 2005). Pendant 2-cyanoethyl groups attached to the [9]aneN_3_ framework are not involved in the coordination to the Ni^II^ center and point away from the macrocyclic cavity.

Two coordinated Cl^-^ anions participate in the formation of multiple C—H···Cl hydrogen bonds (Table 2), which serve to link the complexes into two-dimensional sheets parallel to (010). These sheets are further connected through C—H···N hydrogen bonds, generating a three-dimensional supramolecular network (Fig. 2).

### Experimental

The triazamacrocyclic ligand 1,4,7-tris(2-cyanoethyl)-1,4,7-triazacyclononane was prepared following a literature procedure (Tei *et al.*, 1998). A mixture of the triazamacrocyclic ligand (29 mg, 0.1 mmol) and NiCl_2_.6H_2_O (24 mg, 0.1 mmol) in MeOH (10 ml) was refluxed for 2 h. The precipitated green solid was filtered off and subsequently dissolved in dimethylformamide. Green single crystals of (I) suitable for X-ray diffraction analysis were obtained by slow diffusion of diethyl ether into the dimethylformamide solution. (yield: 31 mg, 63.2%). Analysis: found C 43.87, H 6.53, N 20.04%; calculated for C_18_H_31_Cl_2_N_7_NiO C 44.02, H 6.36, N 19.97%.

### Refinement

All H atoms were placed in calculated positions and treated in the subsequent refinement as riding atoms, with C—H distances in the range 0.93–0.97 Å and *U*_iso_(H) = 1.2 *U*_eq_(C) or 1.5 *U*_eq_(methyl C).

### Crystal data

**Table:**

[NiCl_2_(C_15_H_24_N_6_)(C_3_H_7_NO)]	*F*_000_ = 1032
*M**_r_* = 491.11	*D*_x_ = 1.385 Mg m^−^^3^
Monoclinic, *P*2_1_/*n*	Mo *K*α radiation λ = 0.71073 Å
Hall symbol: -P 2yn	Cell parameters from 2569 reflections
*a* = 9.7657 (10) Å	θ = 2.4–23.0º
*b* = 19.698 (2) Å	µ = 1.07 mm^−^^1^
*c* = 12.3504 (13) Å	*T* = 298 (2) K
β = 97.676 (2)º	Cell measurement pressure: 101(2) kPa
*V* = 2354.5 (4) Å^3^	Block, green
*Z* = 4	0.32 × 0.24 × 0.22 mm

### Data collection

**Table:**

Bruker SMART APEX CCD area-detector diffractometer	4616 independent reflections
Radiation source: sealed tube	3371 reflections with *I* > 2σ(*I*)
Monochromator: graphite	*R*_int_ = 0.050
*T* = 298(2) K	θ_max_ = 26.0º
ω and φ scans	θ_min_ = 2.0º
Absorption correction: multi-scan(SADABS; Bruker, 2000)	*h* = −12→12
*T*_min_ = 0.725, *T*_max_ = 0.798	*k* = −24→19
12684 measured reflections	*l* = −14→15

### Refinement

**Table:**

Refinement on *F*^2^	Secondary atom site location: difference Fourier map
Least-squares matrix: full	Hydrogen site location: inferred from neighbouring sites
*R*[*F*^2^ > 2σ(*F*^2^)] = 0.053	H-atom parameters constrained
*wR*(*F*^2^) = 0.115	*w* = 1/[σ^2^(*F*_o_^2^) + (0.05*P*)^2^ + 1.55*P*] where *P* = (*F*_o_^2^ + 2*F*_c_^2^)/3
*S* = 1.01	(Δ/σ)_max_ < 0.001
4616 reflections	Δρ_max_ = 0.32 e Å^−^^3^
265 parameters	Δρ_min_ = −0.48 e Å^−^^3^
Primary atom site location: structure-invariant direct methods	Extinction correction: none

### Fractional atomic coordinates and isotropic or equivalent isotropic displacement parameters (Å^2^)

**Table:**

	*x*	*y*	*z*	*U*_iso_*/*U*_eq_	
C1	0.2245 (3)	0.82135 (19)	0.3865 (3)	0.0332 (8)	
H1A	0.2389	0.7806	0.4307	0.040*	
H1B	0.2251	0.8599	0.4355	0.040*	
C2	0.3401 (4)	0.8287 (2)	0.3188 (3)	0.0362 (8)	
H2B	0.4277	0.8238	0.3654	0.043*	
H2A	0.3371	0.8738	0.2870	0.043*	
C3	0.3526 (4)	0.70715 (18)	0.2734 (3)	0.0349 (8)	
H3A	0.3479	0.7078	0.3514	0.042*	
H3B	0.4437	0.6912	0.2624	0.042*	
C4	0.2440 (4)	0.65873 (18)	0.2181 (3)	0.0366 (8)	
H4A	0.2621	0.6501	0.1440	0.044*	
H4B	0.2503	0.6158	0.2571	0.044*	
C5	0.0623 (4)	0.69268 (18)	0.3255 (3)	0.0366 (8)	
H5A	0.1427	0.6852	0.3792	0.044*	
H5B	−0.0044	0.6574	0.3349	0.044*	
C6	0.0012 (4)	0.75946 (19)	0.3469 (3)	0.0343 (8)	
H6B	−0.0091	0.7626	0.4238	0.041*	
H6A	−0.0901	0.7628	0.3053	0.041*	
C7	0.0122 (4)	0.88213 (18)	0.3182 (3)	0.0342 (8)	
H7A	−0.0541	0.8841	0.2524	0.041*	
H7B	0.0780	0.9185	0.3136	0.041*	
C8	−0.0650 (4)	0.8969 (2)	0.4150 (3)	0.0414 (9)	
H8A	−0.1106	0.9405	0.4029	0.050*	
H8B	−0.1364	0.8628	0.4164	0.050*	
C9	0.0205 (5)	0.8984 (2)	0.5247 (4)	0.0558 (12)	
C10	0.4296 (4)	0.7914 (2)	0.1522 (3)	0.0368 (8)	
H10A	0.3966	0.8310	0.1097	0.044*	
H10B	0.4280	0.7535	0.1019	0.044*	
C11	0.5811 (4)	0.8044 (2)	0.2007 (3)	0.0421 (9)	
H11A	0.6035	0.7764	0.2651	0.051*	
H11B	0.6408	0.7907	0.1478	0.051*	
C12	0.6089 (4)	0.8739 (2)	0.2298 (4)	0.0464 (10)	
C13	0.0002 (4)	0.64777 (18)	0.1412 (3)	0.0399 (9)	
H13A	0.0219	0.6536	0.0674	0.048*	
H13B	−0.0894	0.6685	0.1440	0.048*	
C14	−0.0142 (5)	0.5712 (2)	0.1616 (4)	0.0497 (10)	
H14A	−0.0132	0.5635	0.2393	0.060*	
H14B	−0.1026	0.5558	0.1247	0.060*	
C15	0.0981 (5)	0.5304 (2)	0.1224 (4)	0.0562 (12)	
C16	0.1301 (4)	0.90640 (19)	0.0180 (3)	0.0386 (9)	
H16	0.0691	0.8790	−0.0269	0.046*	
C17	0.2656 (6)	1.0076 (3)	0.0392 (4)	0.0698 (16)	
H17A	0.2512	1.0069	0.1145	0.105*	
H17B	0.2524	1.0529	0.0111	0.105*	
H17C	0.3580	0.9929	0.0330	0.105*	
C18	0.1201 (6)	0.9843 (3)	−0.1349 (4)	0.0695 (15)	
H18A	0.0661	0.9484	−0.1718	0.104*	
H18B	0.1977	0.9938	−0.1726	0.104*	
H18C	0.0641	1.0243	−0.1339	0.104*	
Cl1	0.17488 (9)	0.74622 (4)	−0.02223 (7)	0.0322 (2)	
Cl2	−0.11545 (8)	0.80646 (4)	0.07904 (7)	0.0317 (2)	
N1	0.0873 (3)	0.81727 (13)	0.3173 (2)	0.0270 (6)	
N2	0.3315 (3)	0.77684 (15)	0.2292 (2)	0.0330 (7)	
N3	0.1036 (3)	0.68642 (15)	0.2155 (2)	0.0329 (6)	
N4	0.0800 (4)	0.8977 (2)	0.6093 (3)	0.0572 (10)	
N5	0.6267 (4)	0.9299 (2)	0.2485 (3)	0.0616 (11)	
N6	0.1815 (4)	0.4987 (2)	0.0903 (3)	0.0617 (11)	
N7	0.1697 (4)	0.96334 (18)	−0.0217 (3)	0.0538 (10)	
Ni1	0.12289 (5)	0.78712 (2)	0.15350 (4)	0.03039 (14)	
O1	0.1693 (3)	0.88679 (12)	0.11226 (19)	0.0358 (6)	

### Atomic displacement parameters (Å^2^)

**Table:**

	*U*^11^	*U*^22^	*U*^33^	*U*^12^	*U*^13^	*U*^23^
C1	0.0328 (19)	0.042 (2)	0.0252 (18)	0.0027 (15)	0.0034 (14)	−0.0026 (15)
C2	0.0270 (17)	0.048 (2)	0.032 (2)	0.0031 (15)	−0.0006 (14)	−0.0051 (16)
C3	0.0298 (18)	0.042 (2)	0.0324 (19)	0.0158 (15)	0.0016 (14)	0.0045 (16)
C4	0.054 (2)	0.0288 (18)	0.0279 (19)	0.0061 (16)	0.0081 (16)	0.0058 (15)
C5	0.048 (2)	0.0265 (18)	0.037 (2)	−0.0006 (15)	0.0106 (17)	0.0025 (15)
C6	0.0291 (18)	0.040 (2)	0.034 (2)	−0.0007 (15)	0.0077 (15)	0.0037 (16)
C7	0.043 (2)	0.0294 (19)	0.0311 (19)	0.0040 (15)	0.0103 (16)	−0.0047 (15)
C8	0.045 (2)	0.043 (2)	0.038 (2)	0.0070 (17)	0.0135 (17)	−0.0058 (17)
C9	0.068 (3)	0.063 (3)	0.042 (3)	−0.014 (2)	0.027 (2)	−0.018 (2)
C10	0.0333 (19)	0.050 (2)	0.0278 (19)	−0.0001 (16)	0.0079 (15)	−0.0020 (16)
C11	0.0308 (19)	0.066 (3)	0.032 (2)	0.0045 (18)	0.0121 (16)	0.0035 (18)
C12	0.031 (2)	0.049 (3)	0.061 (3)	−0.0012 (18)	0.0136 (18)	0.002 (2)
C13	0.041 (2)	0.029 (2)	0.048 (2)	−0.0078 (16)	0.0020 (17)	0.0043 (16)
C14	0.057 (3)	0.040 (2)	0.054 (3)	−0.0087 (19)	0.013 (2)	−0.0047 (19)
C15	0.049 (3)	0.042 (2)	0.075 (3)	0.004 (2)	−0.003 (2)	−0.021 (2)
C16	0.043 (2)	0.039 (2)	0.035 (2)	−0.0012 (16)	0.0080 (17)	0.0145 (16)
C17	0.079 (4)	0.061 (3)	0.061 (3)	−0.036 (3)	−0.020 (3)	0.020 (2)
C18	0.074 (3)	0.068 (3)	0.062 (3)	−0.012 (3)	−0.006 (3)	0.035 (3)
Cl1	0.0318 (4)	0.0364 (5)	0.0285 (4)	0.0003 (3)	0.0045 (3)	−0.0005 (3)
Cl2	0.0306 (4)	0.0356 (4)	0.0287 (4)	0.0001 (3)	0.0038 (3)	−0.0004 (3)
N1	0.0264 (14)	0.0266 (14)	0.0287 (15)	0.0040 (11)	0.0064 (11)	0.0023 (11)
N2	0.0238 (15)	0.0357 (17)	0.0401 (18)	−0.0016 (12)	0.0066 (12)	−0.0054 (13)
N3	0.0339 (15)	0.0323 (16)	0.0330 (16)	−0.0026 (12)	0.0057 (12)	0.0018 (13)
N4	0.059 (2)	0.074 (3)	0.041 (2)	−0.003 (2)	0.0162 (18)	−0.0245 (19)
N5	0.058 (2)	0.063 (3)	0.071 (3)	−0.016 (2)	0.034 (2)	−0.020 (2)
N6	0.070 (3)	0.055 (2)	0.060 (3)	0.010 (2)	0.009 (2)	−0.0248 (19)
N7	0.070 (2)	0.045 (2)	0.045 (2)	−0.0092 (18)	0.0022 (18)	0.0168 (17)
Ni1	0.0296 (2)	0.0339 (3)	0.0277 (2)	−0.00010 (18)	0.00394 (17)	0.00023 (19)
O1	0.0419 (14)	0.0377 (14)	0.0267 (13)	−0.0016 (11)	0.0002 (11)	0.0069 (11)

### Geometric parameters (Å, °)

**Table:**

C1—N1	1.492 (4)	C10—H10A	0.9700
C1—C2	1.499 (5)	C10—H10B	0.9700
C1—H1A	0.9700	C11—C12	1.433 (6)
C1—H1B	0.9700	C11—H11A	0.9700
C2—N2	1.500 (5)	C11—H11B	0.9700
C2—H2B	0.9700	C12—N5	1.134 (6)
C2—H2A	0.9700	C13—N3	1.480 (5)
C3—N2	1.482 (5)	C13—C14	1.538 (5)
C3—C4	1.518 (5)	C13—H13A	0.9700
C3—H3A	0.9700	C13—H13B	0.9700
C3—H3B	0.9700	C14—C15	1.491 (6)
C4—N3	1.473 (5)	C14—H14A	0.9700
C4—H4A	0.9700	C14—H14B	0.9700
C4—H4B	0.9700	C15—N6	1.139 (6)
C5—N3	1.473 (5)	C16—O1	1.238 (4)
C5—C6	1.483 (5)	C16—N7	1.304 (5)
C5—H5A	0.9700	C16—H16	0.9300
C5—H5B	0.9700	C17—N7	1.419 (6)
C6—N1	1.490 (4)	C17—H17A	0.9600
C6—H6B	0.9700	C17—H17B	0.9600
C6—H6A	0.9700	C17—H17C	0.9600
C7—N1	1.474 (4)	C18—N7	1.476 (6)
C7—C8	1.524 (5)	C18—H18A	0.9600
C7—H7A	0.9700	C18—H18B	0.9600
C7—H7B	0.9700	C18—H18C	0.9600
C8—C9	1.493 (6)	Cl1—Ni1	2.4315 (10)
C8—H8A	0.9700	Cl2—Ni1	2.4158 (10)
C8—H8B	0.9700	N1—Ni1	2.180 (3)
C9—N4	1.126 (6)	N2—Ni1	2.134 (3)
C10—N2	1.466 (4)	N3—Ni1	2.144 (3)
C10—C11	1.541 (5)	Ni1—O1	2.093 (2)
			
N1—C1—C2	111.8 (3)	C14—C13—H13A	107.8
N1—C1—H1A	109.3	N3—C13—H13B	107.8
C2—C1—H1A	109.3	C14—C13—H13B	107.8
N1—C1—H1B	109.3	H13A—C13—H13B	107.1
C2—C1—H1B	109.3	C15—C14—C13	112.9 (4)
H1A—C1—H1B	107.9	C15—C14—H14A	109.0
C1—C2—N2	111.9 (3)	C13—C14—H14A	109.0
C1—C2—H2B	109.2	C15—C14—H14B	109.0
N2—C2—H2B	109.2	C13—C14—H14B	109.0
C1—C2—H2A	109.2	H14A—C14—H14B	107.8
N2—C2—H2A	109.2	N6—C15—C14	178.3 (5)
H2B—C2—H2A	107.9	O1—C16—N7	123.5 (4)
N2—C3—C4	111.2 (3)	O1—C16—H16	118.2
N2—C3—H3A	109.4	N7—C16—H16	118.2
C4—C3—H3A	109.4	N7—C17—H17A	109.5
N2—C3—H3B	109.4	N7—C17—H17B	109.5
C4—C3—H3B	109.4	H17A—C17—H17B	109.5
H3A—C3—H3B	108.0	N7—C17—H17C	109.5
N3—C4—C3	111.7 (3)	H17A—C17—H17C	109.5
N3—C4—H4A	109.3	H17B—C17—H17C	109.5
C3—C4—H4A	109.3	N7—C18—H18A	109.5
N3—C4—H4B	109.3	N7—C18—H18B	109.5
C3—C4—H4B	109.3	H18A—C18—H18B	109.5
H4A—C4—H4B	107.9	N7—C18—H18C	109.5
N3—C5—C6	113.9 (3)	H18A—C18—H18C	109.5
N3—C5—H5A	108.8	H18B—C18—H18C	109.5
C6—C5—H5A	108.8	C7—N1—C6	111.3 (3)
N3—C5—H5B	108.8	C7—N1—C1	111.0 (3)
C6—C5—H5B	108.8	C6—N1—C1	113.2 (3)
H5A—C5—H5B	107.7	C7—N1—Ni1	112.6 (2)
C5—C6—N1	112.3 (3)	C6—N1—Ni1	100.7 (2)
C5—C6—H6B	109.1	C1—N1—Ni1	107.69 (19)
N1—C6—H6B	109.1	C10—N2—C3	110.5 (3)
C5—C6—H6A	109.1	C10—N2—C2	111.6 (3)
N1—C6—H6A	109.1	C3—N2—C2	111.5 (3)
H6B—C6—H6A	107.9	C10—N2—Ni1	111.5 (2)
N1—C7—C8	118.1 (3)	C3—N2—Ni1	109.1 (2)
N1—C7—H7A	107.8	C2—N2—Ni1	102.4 (2)
C8—C7—H7A	107.8	C4—N3—C5	112.3 (3)
N1—C7—H7B	107.8	C4—N3—C13	112.3 (3)
C8—C7—H7B	107.8	C5—N3—C13	111.5 (3)
H7A—C7—H7B	107.1	C4—N3—Ni1	103.0 (2)
C9—C8—C7	116.1 (3)	C5—N3—Ni1	107.4 (2)
C9—C8—H8A	108.3	C13—N3—Ni1	109.9 (2)
C7—C8—H8A	108.3	C16—N7—C17	122.5 (4)
C9—C8—H8B	108.3	C16—N7—C18	121.4 (4)
C7—C8—H8B	108.3	C17—N7—C18	116.1 (4)
H8A—C8—H8B	107.4	O1—Ni1—N2	88.31 (10)
N4—C9—C8	176.6 (5)	O1—Ni1—N3	170.81 (11)
N2—C10—C11	117.2 (3)	N2—Ni1—N3	83.12 (11)
N2—C10—H10A	108.0	O1—Ni1—N1	92.12 (10)
C11—C10—H10A	108.0	N2—Ni1—N1	83.15 (11)
N2—C10—H10B	108.0	N3—Ni1—N1	83.59 (11)
C11—C10—H10B	108.0	O1—Ni1—Cl2	89.34 (7)
H10A—C10—H10B	107.2	N2—Ni1—Cl2	175.06 (8)
C12—C11—C10	113.4 (3)	N3—Ni1—Cl2	98.94 (8)
C12—C11—H11A	108.9	N1—Ni1—Cl2	92.60 (8)
C10—C11—H11A	108.9	O1—Ni1—Cl1	90.90 (7)
C12—C11—H11B	108.9	N2—Ni1—Cl1	93.35 (8)
C10—C11—H11B	108.9	N3—Ni1—Cl1	92.91 (8)
H11A—C11—H11B	107.7	N1—Ni1—Cl1	175.31 (8)
N5—C12—C11	176.7 (5)	Cl2—Ni1—Cl1	91.02 (3)
N3—C13—C14	118.2 (3)	C16—O1—Ni1	118.2 (2)
N3—C13—H13A	107.8		

### Hydrogen-bond geometry (Å, °)

**Table:**

*D*—H···*A*	*D*—H	H···*A*	*D*···*A*	*D*—H···*A*
C1—H1A···Cl2^i^	0.97	2.76	3.667 (4)	156
C2—H2A···O1	0.97	2.54	3.076 (5)	115
C3—H3A···Cl2^i^	0.97	2.80	3.756 (4)	168
C7—H7A···Cl2	0.97	2.63	3.394 (4)	135
C10—H10A···O1	0.97	2.48	3.147 (5)	126
C10—H10B···Cl1	0.97	2.73	3.192 (4)	110
C11—H11A···Cl1^i^	0.97	2.66	3.565 (4)	155
C11—H11B···Cl2^ii^	0.97	2.65	3.497 (4)	146
C13—H13A···Cl1	0.97	2.69	3.415 (4)	132
C16—H16···Cl1	0.93	2.81	3.233 (4)	109
C16—H16···Cl2	0.93	2.76	3.268 (4)	115
C18—H18B···N5^iii^	0.96	2.55	3.445 (7)	155

Symmetry codes: (i) *x*+1/2, −*y*+3/2, *z*+1/2; (ii) *x*+1, *y*, *z*; (iii) −*x*+1, −*y*+2, −*z*.

## References

[bb1] Bruker (2000). *SMART*, *SAINT* and *SADABS* Bruker AXS Inc., Madison, Wisconsin, USA.

[bb2] Farrugia, L. J. (1997). *J. Appl. Cryst.* **30**, 565.

[bb3] Graham, B., Spiccia, L., Batten, S. R., Skelton, B. W. & White, A. H. (2005). *Inorg. Chim. Acta*, **358**, 3983–3994.

[bb4] Li, Q.-X., Luo, Q.-H., Li, Y.-Z., Duan, C.-Y. & Tu, Q.-Y. (2005). *Inorg. Chim. Acta*, **358**, 504–512.

[bb5] Schlager, O., Wieghardt, K., Grondey, H., Rufinska, A. & Nuber, B. (1995). *Inorg. Chem.* **34**, 6440–6448.

[bb6] Sheldrick, G. M. (2008). *Acta Cryst.* A**64**, 112–122. 10.1107/S0108767307043930 18156677

[bb7] Tei, L., Blake, A. J., Lippolis, V., Wilson, C. & Schröder, M. (2003). *J. Chem. Soc. Dalton Trans.* pp. 304–310.

[bb8] Tei, L., Lippolis, V., Blake, A. J., Cooke, P. A. & Schröder, M. (1998). *Chem. Commun.* pp. 2633–2634.

[bb9] Zhang, Z., Geng, Z.-R., Zhao, Q. & Wang, Z.-L. (2008). *Acta Cryst.* E**64**, m1041–m1042. 10.1107/S160053680802179X PMC2961961 21203031

